# Let-7a-5p Regulates Animal Lipid Accumulation by Targeting Srebf2 and Thbs1 Signaling

**DOI:** 10.3390/ijms25020894

**Published:** 2024-01-11

**Authors:** Jiahao Shao, Genglong Jiang, Yanhong Li, Meigui Wang, Tao Tang, Jie Wang, Xianbo Jia, Songjia Lai

**Affiliations:** College of Animal Science and Technology, Sichuan Agricultural University, Chengdu 611130, China; shaojh1997@163.com (J.S.); jianggl1196282249@163.com (G.J.); lyh81236718@163.com (Y.L.); wmg1987797495@163.com (M.W.); m18483220592@163.com (T.T.); wjie68@163.com (J.W.); jaxb369@sicau.edu.cn (X.J.)

**Keywords:** lipid accumulation, high-fat diet, preadipocytes, white adipose tissue

## Abstract

Recently, the trend of obesity is becoming increasingly prevalent, and the underlying pathogenesis of obesity is complex and needs to be researched further. In this study, we report a decreased expression of let-7a-5p in the white adipose tissue (WAT) of animals with obesity. Using the RNA oligo, let-7a-5p over-expression or suppression–expression is achieved, impacting the proliferation and differentiation of preadipocytes in vitro. *Srebf2* mechanistically interacts with the metabolic effect of let-7a-5p and participates in lipid accumulation by regulating *Srebf2* downstream signaling. Moreover, let-7a-5p binds to *Thbs1* to interact with the PI3K-AKT-mTOR pathway, down-regulating the phosphorylation levels of AKT, mTOR, and S6K1 to decrease lipid accumulation. In conclusion, our study highlights the physiological significance of let-7a-5p in lipid accumulation and suggests that the let-7a-5p/Srebf2 and let-7a-5p/Thbs1/PI3K-AKT-mTOR axes may represent potential mechanisms for controlling lipid accumulation in obesity.

## 1. Introduction

Obesity is defined as an excessive body mass index (BMI) that is associated with white adipose tissue (WAT) accumulation, as well as an increased risk of endocrine disease, type II diabetes, and cancer [1,2,3,4]. WAT is a multi-depot organ that supports endocrine and energy storage. WAT has the unique ability to coordinate metabolic changes throughout the body and integrate responses to maintain metabolic homeostasis [5,6]. Thus, the maintenance of WAT lipid metabolism is essential. However, comprehending the pathological process of fat buildup is difficult because both environment and genes influence lipid metabolic regulation [7]. Nowadays, highly palatable and fat-dense foods are becoming more and more available and cheaper, and this, along with the development of food production, processing, storage, and preparation, contributes to the rise in obesity [8]. Furthermore, extensive data have indicated that multiple genes, gene products, and signaling pathways are significant contributors to the control of WAT metabolism. Overall, 15–50% of the cells in the WAT are preadipocytes [9]. During adipogenesis, preadipocyte transcription factors such as Sox9 and WNT/β-catenine undergo suppression, while the CCAAT/enhancer-binding protein (C/EBP) family of bZIP transcription factors and PPARγ are active [6,10,11]. Furthermore, some micronutrients, such as vitamins, constitute an essential functional element of the development of WAT upon their transformation into hormone-like molecules by certain enzymes [12].

microRNAs (miRNAs) pair with the 3′ untranslated regions (UTR) to regulate the post-transcriptional suppression of target genes. miRNAs usually exhibit cell type-, tissue-, and developmental stage-specific expression and influence intracorporeal metabolic processes. Emerging data highlight the roles of miRNAs in lipid metabolic regulation and the progression of lipid dysmetabolism-related diseases. For instance, in humans and animals, normal and obese individuals differ with respect to adipose miRNA expression [13,14]. miR-143a-3p regulates preadipocyte proliferation and differentiation by targeting MAPK7 [15]. In addition, miR-214-3p promotes the differentiation of 3T3-L1 through the Ctnnb1/Wnt/β-Catenin pathway [16]. Overall, miRNAs have attracted a lot of interest for their involvement in lipid metabolism and diseases. Although major pathways leading to lipid accumulation in adipocytes have been identified, there are still many roles for miRNAs in the context of obesity that need to be investigated.

In this study, we discovered a miRNA named let-7a-5p, which is down-regulated in obese individuals, and examined its function further in vivo and in vitro. Our findings indicate that the let-7a-5p/Srebf2 and let-7a-5p/Thbs1/PI3K-AKT-mTOR axes may be crucial in regulating animal lipid accumulation and may contribute to the development of new therapies for obesity.

## 2. Results

### 2.1. Let-7a-5p Is Associated with Lipid Metabolism

**Figure 1:**
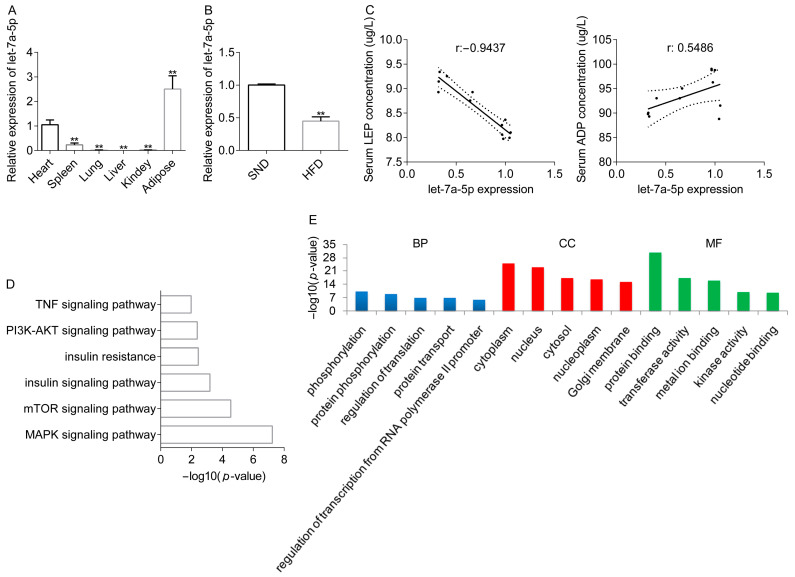
Let-7a-5p is associated with lipid metabolism. (**A**) Let-7a-5p expression levels in the different organs from the SND group were measured (*n* = 6). (**B**) Let-7a-5p expression was assessed in the rabbits’ WAT, and the data based on the normal rabbits were used as a reference (*n* = 6). (**C**) The Pearson correlation between let-7a-5p expression in the rabbits’ WAT and serum LEP (**left**) or ADP (**right**) levels (*n* = 12 in biological animals). (**D**) The pathways partly enriched in the KEGG database. (**E**) A list of the top five important GO terms in MF, CC, and BP. The data are presented as means ± SEM. ** *p*-value < 0.01.

miRNAs are conserved among species with distinct temporal and geographical patterns of expression in response to many biological activities [17,18]. We sequenced the miRNA expression in the adipose tissue from the SND and HFD groups to discover miRNAs that may play a function in lipid metabolism by binding protein-coding genes in the adipose tissue [19]. Interestingly, we noted that let-7a-5p was markedly decreased and showed a similar alternation in other obese animals [13,20] and obese humans [14]. In this study, we first assessed the abundance of let-7a-5p in the organs of the SND rabbits, such as the liver, heart, etc., to verify the expression pattern of let-7a-5p in animals with a normal metabolic condition. Compared with the control animals, let-7a-5p expression was observed to be high in the adipose tissue of the obese animals, suggesting that let-7a-5p may be functionally important for preserving the balance of lipid metabolism (Figure 1A). In addition, we found that let-7a-5p was significantly decreased in the WAT of rabbits subjected to the HFD treatment, which validates the sequencing data (Figure 1B). Next, the major hormones secreted from the adipose tissue, serum LEP and ADP, were next measured in the rabbits. We discovered that let-7a-5p expression in the adipose tissue was negatively correlated with serum LEP levels but positively correlated with serum ADP levels (Figure 1C). Using the Starbase database, let-7a-5p targets were captured, including *Srebf2*, *Dagla*, *Ptgs2*, and *Thbs1* (Table S7). For the let-7a-5p target genes, lipid metabolic pathways such as the mTOR signaling pathway, PI3K-AKT signaling pathway, and insulin signaling pathway were enriched in the KEGG database (Figure 1D, Table S8). According to the results of GO analysis, the let-7a-5p target genes were significantly enriched in 787 GO terms (483 biological processes (BP), 153 cellular components (CC), and 151 molecular functions (MF)), including the regulation of cell size, cell cycle, the metabolic process, and the cellular response to insulin stimulus (Figure 1E, Table S9). Taken together, these findings suggest that let-7a-5p may be a functional molecule involved in lipid metabolism.

### 2.2. Let-7a-5p Regulates Adipogenesis by Targeting the PI3K-AKT-mTOR Signaling

**Figure 2:**
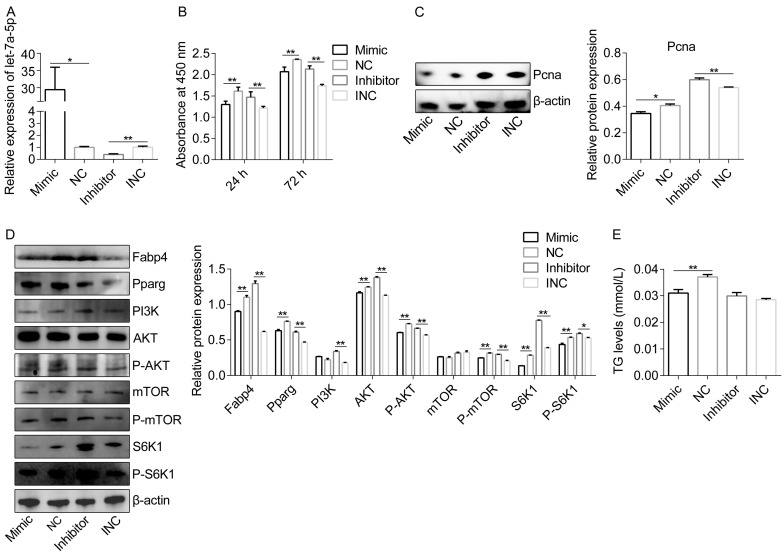
Let-7a-5p regulates adipogenesis by targeting the PI3K-AKT-mTOR signaling. (**A**) The miR-24-3p mimic and inhibitor’s transfection efficiency were assessed (*n* = 6). (**B**) The absorbance of preadipocytes from rabbits transfected with let-7a-5p mimic, NC, let-7a-5p inhibitor, and INC at 24 h and 72 h (*n* = 6). (**C**) Representative Pcna bands in rabbit preadipocytes transfected with let-7a-5p mimic, NC, let-7a-5p inhibitor, and INC. The Pcna expression was quantitatively analyzed (*n* = 3). (**D**) Representative WB of Fabp4, Pparg, PI3K, AKT, P-AKT, mTOR, P-mTOR, S6K1, P-S6K1, and β-actin at 4 days after transfection with the let-7a-5p mimic, NC, let-7a-5p inhibitor, and INC in rabbits’ preadipocytes in the context of differentiation. *n* = 3 for data analysis. (**E**) After treating the preadipocytes with the let-7a-5p mimic, NC, let-7a-5p inhibitor, and INC, the TG content was assessed (*n* = 8). The data are presented as means ± SEM. * *p*-value < 0.05; ** *p*-value < 0.01.

Measuring the effect of let-7a-5p on lipid metabolic capabilities is important since it is connected to several protein-coding genes linked to lipid metabolism and is expressed at low levels in the adipose tissue of obese rabbits. Next, by transfecting the let-7a-5p mimic, NC, let-7a-5p inhibitor, and INC into the preadipocytes, we examined the impact of let-7a-5p on the adipogenesis of rabbit preadipocytes. We verified that the transfected analogs effectively increased or lowered let-7a-5p abundance in vitro (Figure 2A). For preadipocytes proliferation, the let-7a-5p mimic decreased the absorbance to different extents at various lengths of time, but the let-7a-5p inhibitor markedly enhanced absorbance after transfection for 24 h and 72 h (Figure 2B). Following treatment with the let-7a-5p mimic, the expression of the proliferation-related protein Pcna was reduced; however, following treatment with the let-7a-5p inhibitor, it was increased (Figure 2C). Furthermore, we verified that the let-7a-5p mimic, which reversed the effects of the let-7a-5p inhibitor after 4 days of differentiation, decreased the protein levels of Fabp4 and Pparg, which are crucial transcription factors in the development and function of adipose tissue and markers of preadipocyte differentiation (Figure 2D). Additionally, the TG accumulation in the mimic group was less than that in the NC group (Figure 2E). 

Meanwhile, we also wanted to determine whether let-7a-5p is functionally involved in the lipid metabolic regulation of the PI3K-AKT-mTOR pathway. To test this notion, we first confirmed whether let-7a-5p inhibition and over-expression could both significantly change PI3K activity (Figure 2D). Subsequently, the over-expressing of let-7a-5 resulted in the suppression of P-AKT expression, while the let-7a-5p inhibitor caused an increase in P-AKT expression (Figure 2D). The downstream phosphorylated mTOR and S6K1 showed a reduction in the let-7a-5p mimic group but drastically increased after let-7a-5p inhibition when compared with the related reference (Figure 2D). In summary, our findings show that let-7a-5p has a direct interaction with preadipocytes adipogenesis via the PI3K-AKT-mTOR pathway.

### 2.3. RNA-seq Revealed Potential Let-7a-5p Target Genes

**Figure 3:**
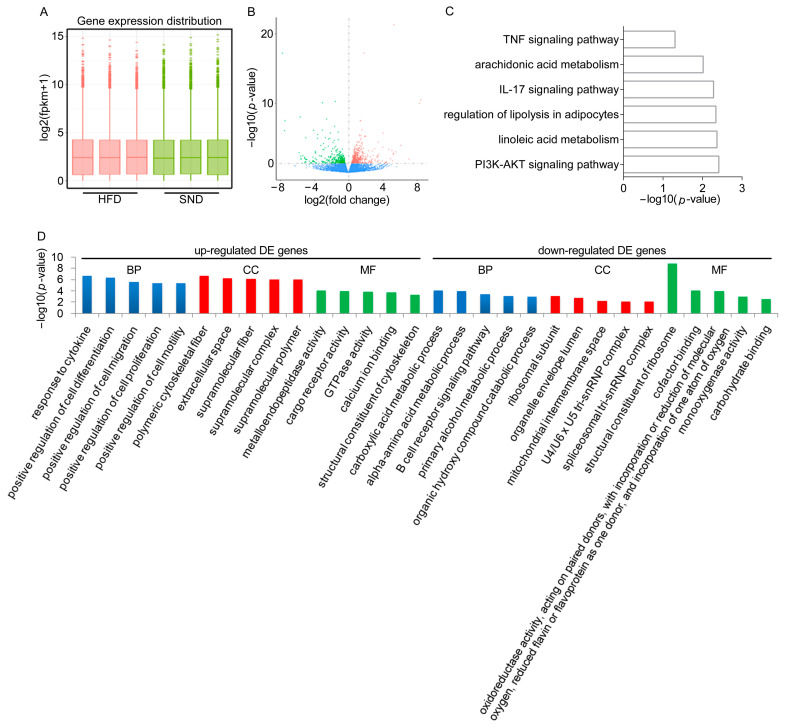
RNA-seq analysis revealed potential let-7a-5p target genes. (**A**) Gene expression distribution from the SND and HFD RNA-seq libraries. (**B**) Based on log2(fold change) and −log10(*p*-value), a volcanic picture of the DE genes between the SND and HFD groups was constructed. The red point indicates the statistically significant up-regulated genes, whereas the green point indicates the statistically significant down-regulated genes. (**C**) The part gene sets enriched in the KEGG database are listed. (**D**) The DE genes enriched in the GO database and only the top five significant GO terms in BP, CC, and MF are listed.

In this study, we performed RNA-seq on adipose RNA collected from the SND and HFD groups to identify the potential let-7a-5p target genes. Overall, six libraries were built successfully, and sequencing for each of the libraries surpassed 45,617,148 high-quality raw reads, with an average clean read of 46,544,061 (Table S1). The Q20 (percentage of reads with a Phred quality value > 20) ranged from 97.77% to 98.07%, and the GC content of libraries ranged from 52.82% to 54.15% (Table S1). Next, the clean data were aligned to reference genomes using HISAT2, with high mapping efficiency ranging from 86.08% to 87.5% (Table S1). The gene expression was quantified using subread software based on read counts. Following the fragments per kilobase per million reads (FPKM) method, we found that the gene expression distribution was consistent between the two metabolic conditions of adipose tissue (Figure 3A).

We identified DE genes using the DESeq following the standard of *p*-value ≤ 0.05. The full list of DE genes is available in Table S2. Among them, 447 genes were down-regulated and 607 genes were up-regulated in the HFD group, respectively (Figure 3B). To better understand the functional roles of DE genes, we subsequently performed an enrichment analysis to categorize the functions of the DE genes in the KEGG database. The data indicated that the PI3K-AKT signaling pathway, IL-17 signaling pathway, arachidonic acid metabolism, linoleic acid metabolism, and TNF signaling pathway were significantly enriched (Figure 3C, Table S3). Meanwhile, based on the up-regulated DE genes, the top significant biological process GO terms are mostly responsible for signaling and cell regulatory functions, such as response to lipoprotein particle, the positive regulation of cell differentiation, the positive regulation of cell migration, the positive regulation of cell proliferation (Figure 3D, Table S4). Genes *Srebf2*, *Lep*, *Ptgs2*, and *Adam17* are important contributors and have been shown to have direct consistency with the above GO terms. Using the down-regulated DE genes, we obtained the related GO terms data (Figure 3D, Table S4). Of the significantly enriched biological process GO terms, the most down-regulated DE genes were shown to control metabolism-related GO terms. Specifically, *Sorl1*, *Lgals12*, and *Pnpla7* are prominently involved in the lipid catabolic process. *Adh2-1*, *Hacl1*, *Echs1*, and *Acad11* are involved in lipid oxidation. Thus, our data revealed a dynamic adipose gene expression under obesity condition. 

### 2.4. Let-7a-5p Regulates Lipid Metabolism by Targeting the Srebf2 Signaling

**Figure 4:**
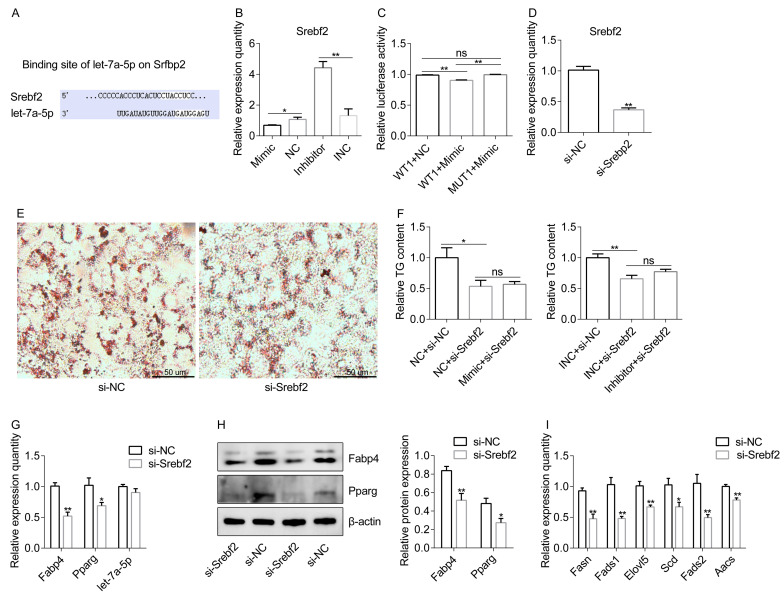
Let-7a-5p regulates lipid metabolism by targeting *Srebf2* signaling. (**A**) Potential binding sites between let-7a-5p and *Srebf2* were identified using the Targetscan website. The sites in the white background represent binding sites. (**B**) The mRNA of *Srebf2* at 4 days after transfection with let-7a-5p mimic, NC, let-7a-5p inhibitor, and INC in the context of differentiation. (*n* = 6). (**C**) Relative luciferase activity was measured after the co-transfection of WT1 and MUT1 with the let-7a-5p mimic and NC, respectively (*n* = 3). (**D**) After si-Srebf2 treatment, the mRNA of *Srebf2* was examined using qRT-PCR (*n* = 6). (**E**) Oil Red O staining was performed for visual observation of the lipid droplets from the si-Srebf2 and si-NC groups. (**F**) The cellular TG concentration was assessed as the indication of lipid accumulation (*n* = 6). (**G**) The expression levels of *Fabp4*, *Pparg*, and let-7a-5p in the 3T3-L1 differentiated mature adipocytes were detected using qRT-PCR (*n* = 6). (**H**) Representative WB of Fabp4 and β-actin at 4 days after transfection with si-Srebf2 and si-NC in the 3T3-L1 preadipocytes in the context of differentiation. *n* = 3 for data analysis. (**I**) qRT-PCR analysis of *Fasn*, *Fads1*, *Elovl5*, *Scd*, *Fads2*, and *Aacs* in the 3T3-L1 preadipocytes treated with si-Srebf2 and si-NC (*n* = 6). Not significant: ns. The data are presented as means ± SEM. * *p*-value < 0.05; ** *p*-value < 0.01.

Based on their sequence information, the Targetscan website was used to determine the possible binding site between *Srebf2* and let-7a-5p (Figure 4A). In terms of preadipocytes differentiation, *Srebf2* mRNA expression was decreased in the mimic group but increased in the inhibitor group in vitro (Figure 4B). Using the luciferase reporter assay, we observed that the luciferase activity of a luc-reporter construct containing *Srebf2* 3′ UTR (WT1) was considerably reduced by the let-7a-5p mimic (Figure 4C). Subsequently, we introduced the specific siRNA target *Srebf2* into cells in vitro to examine the essentialness of *Srebf2* in the metabolic responses of let-7a-5p toward lipid accumulation (Figure 4D). Following si-Srebf2 treatment, 3T3-L1 exhibited reduced lipid accumulation, as demonstrated via Oil Red O staining and intracellular TG analysis, which was consistent with the findings in preadipocytes with let-7a-5p mimic treatment (Figure 4E,F). Importantly, in 3T3-L1 with *Srebf2* knockdown, the further knockdown or overexpression of let-7a-5p did not affect lipid accumulation (Figure 4F). The knockdown of *Srebf2* also reduced the levels of *Fabp4* and *Pparg* genes but did not affect the expression levels of let-7a-5p, implying that *Srebf2* does not modulate the stability of let-7a-5p but might influence the stability of genes related to lipid metabolism (Figure 4G,H). Furthermore, qRT-PCR demonstrated that *Srebf2* knockdown reduced the transcription of genes linked to lipid metabolism, including *Fasn*, *Fads1*, *Elovl5, Scd*, *Fads2*, and *Aacs* (Figure 4I). In conclusion, our findings show that let-7a-5p binds to *Srebf2* and modulates *Srebf2*-mediated lipid metabolic signaling.

### 2.5. A Let-7a-5p Target—Thbs1 Is Required for Adipogenesis via the PI3K-AKT-mTOR Pathway

**Figure 5:**
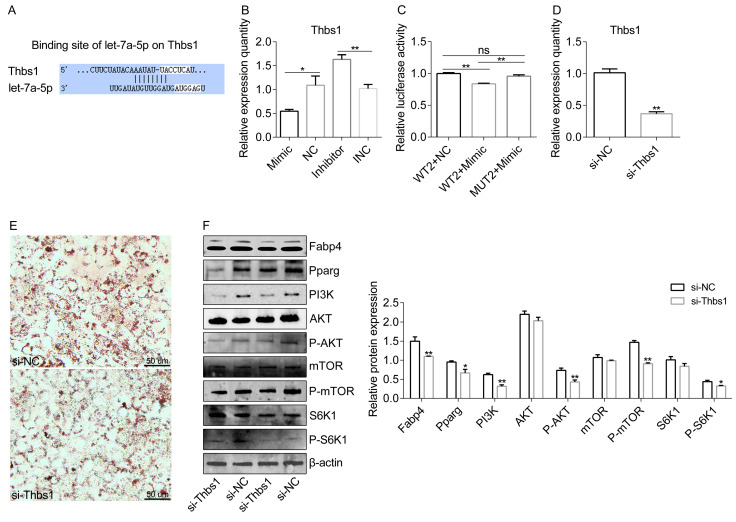
A let-7a-5p target—*Thbs1* is required for adipogenesis via the PI3K-AKT-mTOR pathway. (**A**) Potential binding sites between let-7a-5p and *Thbs1* were identified using the Targetscan website. The sites in the white background represent binding sites. (**B**) The mRNA of *Thbs1* at 4 days after transfection with let-7a-5p mimic, NC, let-7a-5p inhibitor, and INC in the context of differentiation. (*n* = 6). (**C**) Relative luciferase activity was measured after the co-transfection of WT2 and MUT2 with the let-7a-5p mimic and NC, respectively (*n* = 3). (**D**) After si-Thbs1 treatment, the mRNA of *Thbs1* was examined using qRT-PCR (*n* = 6). (**E**) Oil Red O staining was performed for visual observation of the lipid droplets from the si-Thbs1 and si-NC groups. (**F**) Representative bands for Fabp4, Pparg, PI3K, AKT, P-AKT, mTOR, P-mTOR, S6K1, P-S6K1, and β-actin in 3T3-L1 cells treated with si-Thbs1 and si-NC. *n* = 3 for data analysis. Not significant: ns. The data are presented as means ± SEM. * *p*-value < 0.05; ** *p*-value < 0.01.

Considering that let-7a-5p has a direct interaction with adipogenesis via the PI3K-AKT-mTOR pathway, we investigated whether a key gene acts as the mediator. We were intrigued by the identification of *Thbs1* as a putative let-7a-5p target, a functional gene linked to obesity, lipid metabolism, and adipose biology (Figure 5A). *Thbs1* is up-regulated in adipose tissue under obesity (Section 2.3), and in this study, it was negatively associated with let-7a-5p expression (Figure 5B). The luciferase activity of a luc-reporter construct incorporating *Thbs1* 3′ UTR (WT2) was shown to be greatly reduced by the let-7a-5p mimic, but this reduction was abolished by a mutation in the *Thbs1* binding site in the 3′ UTR (Figure 5C). Next, we delivered the specific siRNA target *Thbs1* into cells in vitro to investigate *Thbs1*’s essentialness in the metabolic responses of let-7a-5p toward lipid metabolism (Figure 5D). We noted that the si-Thbs1 group showed lower lipid accumulation, as determined via Oil Red O staining (Figure 5E). Proteins Fabp4 and Pparg were consistently down-regulated after si-Thbs1 treatment (Figure 5F). Meanwhile, the abundance of PI3K protein markedly decreased in the si-Thbs1 group compared with the si-NC group (Figure 5F). Decreased levels of P-AKT were observed, with no significant effect on the total AKT levels after si-Thbs1 treatment (Figure 5F). Moreover, the phosphorylation levels of the downstream mTOR and S6K1 showed a significant decrease, indicating that mTOR signaling was inhibited when *Thbs1* expression was silenced (Figure 5F). These data indicate that *Thbs1* is required for adipogenesis via the PI3K-AKT-mTOR pathway.

### 2.6. Let-7a-5p Inhibites High-Fat-Diet-Induced Obesity in a Mice Model

**Figure 6:**
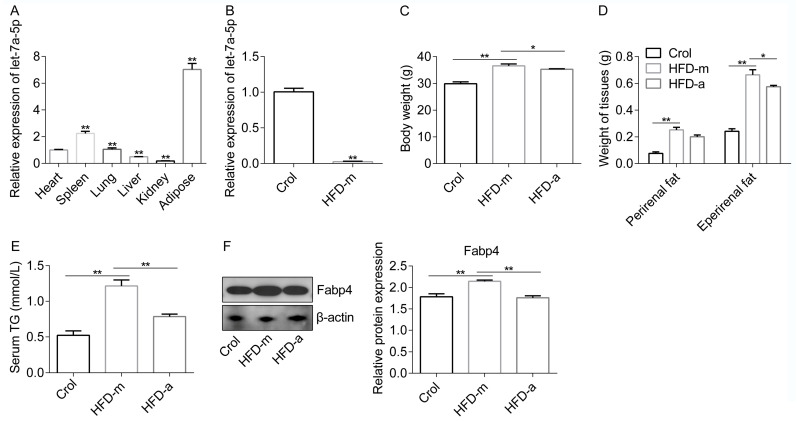
Let-7a-5p inhibits high-fat-diet-induced obesity in a mice model. (**A**) Let-7a-5p expression in the different organs from the Crol group was measured (*n* = 6). (**B**) Let-7a-5p expression was measured in the Crol and HFD-m groups, and the data in the Crol were used as a reference (*n* = 6). Analyses of the metabolic performances of the Crol, HFD-m, and HFD-a groups were carried out, considering body weight (**C**), fat tissue weight (**D**), serum TG abundance (**E**), and lipid metabolism-related protein in the perirenal fat (**F**). *n* = 6 in (**C**–**E**). *n* = 3 in (**F**). The data are presented as means ± SEM. * *p*-value < 0.05; ** *p*-value < 0.01.

At first, we discovered that let-7a-5p was strongly expressed in the adipose tissue of normal metabolic mice, but this was considerably reduced when the mice were fed a high-fat diet, which was consistent with the findings in the HFD rabbit model (Figure 6A,B). Next, we investigated whether let-7a-5p could control lipid accumulation in vivo. We subjected the mice to a high-fat diet and injected them with specific let-7a-5p agomir or agomir control in the caudal vein. After a detailed analysis, it was found that the HFD-a mice featured decreased adiposity compared with the HFD-m mice, as shown by their reduced body weight and adipose tissue weight (Figure 6C,D). Levels of serum TG, which is a marker of lipid metabolism and obesity, were significantly enhanced in the HFD-m group, but let-7a-5p agomir decreased the TG in the HFD-a group compared to the HFD-m group (Figure 6E). At the molecular levels, we examined the expression levels of lipogenesis-related protein Fabp4 in the perirenal adipose tissue of each group. As expected, its expression was decreased after consistent let-7a-5p agomir treatment in the HFD-a group compared to the HFD-m group (Figure 6F). Thus, our data indicate that let-7a-5p may play an important role in the regulation of lipid accumulation in vivo.

## 3. Discussion

Obesity is defined by the World Health Organization (WHO) as the excessive accumulation of WAT. Therefore, it would be beneficial to better understand the molecular network and mechanism of lipid metabolism, including the roles played by miRNAs in effectively controlling lipid metabolism and preventing lipid accumulation in WAT.

Numerous studies have demonstrated that adipose miRNAs exhibit dynamic regulation in obesity and functional roles in adipogenesis [13,15]. For example, miR-24-3p inhibited preadipocytes differentiation, whereas miR-9-5p promoted preadipocytes differentiation [21,22]. According to data available online, let-7a-5p is a key miRNA with roles in bone marrow mesenchymal stem cell (BMSC) osteogenesis [23], embryo implantation [24], shoulder stiffness [25], and inflammatory response [26]. Furthermore, let-7a-5p can decrease *BZW2* expression, and it inhibits hepatoma cell proliferation, invasion, and migration and increases apoptosis [27]. Meanwhile, an oncogenic role for let-7a-5p in colorectal cancer [28] and cervical cancer [29] has been reported. However, although let-7a-5p was markedly decreased in the WAT of obese animals [13,20], its possible role in the adipogenesis and hypertrophy of adipose tissue requires further investigation. In this study, we confirmed that let-7a-5p maintained a low abundance in the WAT of obese rabbits and mice. Gain- or loss-of-function studies showed that let-7a-5p inhibited preadipocytes proliferation and differentiation in vitro. In addition, our data indicate that the over-expression of let-7a-5p altered high-fat-diet-induced body weight, tissue weight gain, and blood fat in the obese mice, suggesting that boosting the expression of let-7a-5p in the obese mice WAT was beneficial for maintaining lipid metabolic homeostasis.

Lipid accumulation is composed of many enzymatic reactions and controlled by a variety of molecules. In this study, targeting research showed that let-7a-5p is directly bound to the 3′-UTR of *Srebf2*. The Srebf2 protein is a member of the sterol regulatory element binding protein (SREBP) nuclear transcription factor family, which regulates lipid homeostasis by controlling the expression of several enzymes necessary for endogenous phospholipid, triacylglycerol, cholesterol, and fatty acid (FA) synthesis [30]. Lipid metabolism is influenced by mature forms of SREBPs that are transcriptionally active and transported to the nucleus, where they bind to the promoters of the SREBP target [31]. Srebp2 promotes the expression of target genes in the cholesterol biosynthesis pathway by binding to the sterol regulator and promoter/enhancer in the lipid synthase gene [32]. In this study, we discovered that *Srebf2* has a direct interaction with the lipid accumulation effect of let-7a-5p via downstream metabolic genes such as *Fasn*, *Fads1*, and *Scd*. Moreover, another possibility is that let-7a-5p regulates lipid accumulation via the PI3K-AKT-mTOR pathway by binding to the 3ʹ UTR of *Thbs1*. Thbs1 is a large adhesive extracellular matrix glycoprotein that contains multiple functional domains. In mice and humans, *Thbs1* is highly expressed in hypertrophic visceral adipose tissue [33,34]. *Thbs1*-null mice were protected against insulin resistance and the inflammation of adipose tissue [35]. Moreover, *Thbs1* deficiency prevents high-fat-diet-induced adipose tissue hypertrophy [33]. We discovered that let-7a-5p regulates the expression of *Thbs1* and that si-Thbs1 inhibits lipid accumulation by targeting the PI3K-AKT-mTOR pathway, indicating the potential connection between let-7a-5p and the metabolic network. It has been revealed that the PI3K-AKT-mTOR pathway controls lipid accumulation in animal cells by increasing the unclear accumulation of mature SREBP [36,37].

However, we cannot exclude the notion that let-7a-5p may cooperate with other actors through unidentified ways to regulate lipid accumulation. Therefore, further mechanistic studies are required to elucidate how let-7a-5p controls lipid accumulation. For instance, it is necessary to investigate the roles that let-7a-5p plays in the process of lipid metabolism in other animal models. It is also necessary to look into the other possible mechanisms of let-7a-5p and their effects on lipid metabolism, such as enhanced lipid secretion through the skin, WAT browning, and metabolomics.

## 4. Materials and Methods

### 4.1. Animals

This work is an extension of a previous study wherein an obese rabbit model was established using a high-fat diet [38]. Briefly, the rabbits in the model were subjected to a control (120 g/d, labeled as the SND group) or a high-fat diet (120 g/d, labeled as the HFD group) for 5 weeks in a thermostatic room. At the end of the trial, six rabbits from each group were selected for sampling, and three samples from each group were used for transcriptome sequencing. Next, eighteen 21-day male Kunming mice were divided into three groups. Two groups received either a normal diet (referred to as the Crol group) or a high-fat diet (referred to as the HFD-m group) for four weeks and received a total of four tail vein injections of agomir control (RiboBio, Guangzhou, China). Moreover, the remaining group was fed a high-fat diet and given four tail vein injections of let-7a-5p agomir (HFD-a group; RiboBio, Guangzhou, China) over four weeks. Every mouse had unrestricted access to food and water. All experimental protocols were performed under the direction of the Institutional Animal Care and Use Committee from the College of Animal Science and Technology, Sichuan Agricultural University, China.

### 4.2. Cell Culture

Cells were kept in a growth medium (GM) containing 10% fetal bovine serum (FBS; Gibco, Grand Island, NY, USA), which was stored in an incubator (Thermo Fisher Scientific, Waltham, MA, USA) at 37 °C and 5% CO_2_ environment. We used a differentiation medium (DM) containing Dulbecco’s modified Eagle’s medium (DMEM; VivaCell, Shanghai, China) supplemented with 10% FBS, 1 μmol/L dexamethasone (DEX; Solarbio, Beijing, China), 0.5 mmol/L 3-isobutyl-1-methylxanthine (IBMX; Solarbio, Beijing, China), and 10 μg/mL insulin (Solarbio, Beijing, China). The maintenance medium (MM) consisted of DMEM with 10% FBS and 10 μg/mL insulin. According to the manufacturer’s instructions, transfection was carried out using the lipofectamine 3000 reagent (Invitrogen, Carlsbad, CA, USA). The transfection reagent was mixed with RNA oligo (let-7a-5p mimic, inhibitor, negative control (NC), inhibitor negative control (INC)) and siRNA (si-Srebf2, si-Thbs1, and si-NC), and then the mixture was transfected into the cells. The concentration of transfection was 50 uM for the mimic and NC, and 100 uM for the inhibitor, INC, siRNA, and si-NC. The above RNA oligo and siRNA were purchased from Sangon Biotech Co., Ltd. (Shanghai, China) and Tsingke Biotechnology Co., Ltd. (Beijing, China), respectively, and Table S5 displays the sequence information.

### 4.3. Quantitative Real-Time Polymerase Chain Reaction (qRT-PCR)

Using the TRIpure reagent (Aidlab, Beijing, China), total RNA was extracted in accordance with the instructions. The quality test was conducted using an Agilent Bioanalyzer (Agilent Technologies, Santa Clara, CA, USA), and only eligible RNA was used for subsequent experiments. For the reverse-transcription of mRNAs and miRNAs, we used the RT Easy^TM^ II (with gDNase) (FOREGENE, Chengdu, China) and the Mir-X^TM^ miRNA First-Strand Synthesis Kit (Takara, Dalian, China), respectively. Furthermore, the NovoStart^®^SYBR qPCR SuperMix Plus (Novoprotein, Shanghai, China) was used in triplicate for qRT-PCR on a CFX96 instrument (Bio-Rad, Hercules, CA, USA). The relative abundance was determined using the 2^−ΔΔCt^ method. U6 was utilized as an internal reference, and the mRQ 3′ primer from the Mir-X^TM^ miRNA First-Strand Synthesis Kit (Takara, Dalian, China) was utilized as the reverse primer for miRNA quantification. Furthermore, the internal reference for mRNA quantification was *β-actin* gene. Table S6 provides a detailed list of primer sequence information.

### 4.4. Western Blotting (WB)

Total protein extraction was carried out using the ProteinExt^®^ Mammalian Total Protein Extraction Kit (TransGen Biotech, Beijing, China). The Bradford protein assay kit (Novoprotein, Shanghai, China) was then used to measure the protein concentration. Briefly, the denatured protein was resolved on the SurePAGE gels (GenScript Corporation, Nanjing, China) and then transferred to the PVDF membrane, which was then sealed with a sealing liquid. The membranes were initially incubated with the corresponding primary antibodies at 4 °C for 8 h. After that, they were incubated with the secondary antibodies (Goat Anti-Rabbit or Anti-Mouse IgG H&L (HRP), Zen Bioscience, Chengdu, China) for 1 h. The antibodies we used are as follows: Pcna (10205-2-AP), purchased from the Proteintech (Wuhan, China); Pparg (A0270) and Fabp4 (A0232), purchased from the Abclonal (Wuhan, China); and AKT (382804), P-AKT (310021), PI3K (251221), mTOR (380411), P-mTOR (381557), S6K1 (R25650), P-S6K1 (380880), and β-actin (380624), purchased from Zen Bioscience (Chengdu, China). Finally, the membranes were exposed to a SuperBright Subpico ECL reagent (SUDGEN, Nanjing, China). Images were taken using a GelDoc system (Bio-Rad, Hercules, CA, USA).

### 4.5. Oil Red O Staining

The cells were fixed for 30 min at room temperature in 4% paraformaldehyde. Subsequently, Oil Red O (Servicebio, Wuhan, China) was mixed with deionized water (3:2) and then added to the cells for 15 min. Ultimately, PBS was used to clean the aforementioned cells until no visible impurities remained. Images were captured using an inverted microscope (Olympus, Tokyo, Japan).

### 4.6. Measuring Blood Markers

All mice were fasted overnight before slaughter and anesthetized via an intraperitoneal injection of chloral hydrate. Blood was collected from the heart and then centrifuged at 4 °C for 5 min to obtain the serum. The commercial enzymatic colorimetry kit (Jiancheng, Nanjing, China) was utilized to test the triglyceride (TG) concentration of the sample following established protocols. In addition, following the standard method, the rabbits’ leptin (LEP) and adiponectin (ADP) levels were tested using a commercial enzyme-linked immunosorbent assay (ELISA) kit (Shanghai Hengyuan Biotechnology Co., Ltd., Shanghai, China).

### 4.7. Measuring Cell Proliferation

When the cell density reached 50%, transfection was carried out. Six hours later, the medium was changed to GM. A cell counting kit (CCK, Zomanbio, Beijing, China) was employed to examine the effect of let-7a-5p on cell proliferation. Briefly, after transfection for 24 h and 72 h, 10 μL CCK reagent was added to each well and incubated at 37 °C and 5% CO_2_ for 2 h. The absorbance, which indicates the quantity of living cells, was then measured using a microplate reader (Thermo Fisher Scientific, Waltham, MA, USA).

### 4.8. Dual-Luciferase Reporter Assay

In this study, we predicted possible let-7a-5p targets using the Starbase database (https://starbase.sysu.edu.cn/, accessed on 10 July 2022). The software DAVID 6.7 (http://david.abcc.ncifcrf.gov/home.jsp, accessed on 18 May 2023) was used to conduct Gene Ontology (GO) analysis and Kyoto Encyclopedia of Genes and Genomes (KEGG) pathway enrichment analysis. To validate the proposed binding site, wild-type (WT) and mutant (MUT) pmirGLO plasmids of the target sequence were constructed by Tsingke Biotechnology Co., Ltd. (Beijing, China). Next, 24-well plates (NEST Biotechnology, Wuxi, China) were seeded with 293T cells. Using the lipofectamine 3000 reagent (Invitrogen, Carlsbad, CA, USA), the let-7a-5p mimic or NC was co-transfected with specific WT or MUT plasmids. After a day, luciferase activities were measured using the Duo-Lite TM Luciferase Assay System (Vazyme, Nanjing, China) in accordance with the manufacturer’s instructions.

### 4.9. Transcriptome Sequencing

Sample RNA was produced using the above-described method. The M-MuLv reverse transcriptase system was used to create the first strand of cDNA, while DNA polymerase I and dNTPs were used to create the second strand. Following the manufacturer’s instructions, cDNA of 250–300 bp was generated, and PCR amplification was then used to create the cDNA libraries using the NEBNext^®^ Ultra™ RNA Library Prep Kit for Illumina^®^ (New England BioLabs, Ipswich, MA, USA). Novogene Co., Ltd. (Beijing, China) used the Illumina NovaSeq 6000 platform to sequence the cDNA libraries. Additionally, the FASTP program (version 0.19.7) was used to clean the raw data. HISAT2 software 2.0.5 was then used to compare the clean data with the reference genome to determine the read localization information. After that, the transcript read count was determined, and differential expression (DE) analysis was performed using the R package DEseq 1.16.1. The threshold was defined to be *p*-value ≤ 0.05. Using the R ggplot2 tool, the DE genes between groups were displayed graphically.

### 4.10. Statistical Analysis

All data are presented as means ± SEM. Student’s *t*-test was utilized to ascertain group differences in the statistical analysis, which was conducted using GraphPad software 6.04 (GraphPad Software Inc., La Jolla, CA, USA). Moreover, a one-way ANOVA was used for multiple comparisons. Differences were considered statistically significant at *p*-value < 0.05.

## 5. Conclusions

In conclusion, our findings suggest that the let-7a-5p/Srebf2 and let-7a-5p/Thbs1/PI3K-AKT-mTOR axes are crucial for lipid accumulation and that they may represent a potential mechanism for controlling lipid accumulation in obesity.

## Data Availability

The sequencing data are available on the NCBI database at Sequence Read Archive (SRA). The transcriptome sequencing data: SUB13840470.

## Supplementary Materials

The supporting information can be downloaded at: https://www.mdpi.com/article/10.3390/ijms25020894/s1.
