# Supplementary material for: Let-7a-5p Regulates Animal Lipid Accumulation by Targeting Srebf2 and Thbs1 Signaling
Source: Int J Mol Sci. 2024 Jan 11;25(2):894. doi: 10.3390/ijms25020894 (PMC10815625; doi:10.3390/ijms25020894)
Supplement: Supplementary file 1 [file ijms-25-00894-s001.zip › The caption of additional Table.pdf]

Table S1: Summary and quality assessment of the mRNA sequencing data; Table S2: The list of all DE genes between the HFD and SND groups; Table S3: The KEGG analysis of DE genes; Table S4: The GO analysis of DE genes; Table S5: The sequence information of RNA oligo and siRNA; Table S6: The sequence information of primer used for the qRT-PCR analysis; Table S7: The target genes of let-7a-5p predicted by using the Starbase database; Table S8: KEGG analysis of let-5a-5p target genes; Table S9: GO analysis of let-7a-5p target genes.
